# On Flour Albus

**Published:** 1855-11

**Authors:** 


On Fluor Albus.—From the examination of a large number of cases at La Charité, Berlin, Dr. Beigel arrives at the following conclusions:

1. The secretion of the uterus is always slightly alkaline, that of the vagina and urethra acid, and that of the Duverney glands neutral. If the secretion is found sometimes acid and sometimes alkaline, this depends upon whether the uterus or the vagina furnishes the largest share. The discharge is, however, almost always sour, as the acid reaction of the vaginal secretion is too considerable to be neutralized by the uterine fluid. It is impossible to declare the infectious or inocuous character of the discharge from its acidity or alkalescence.

2. Great viscidity (*Zähigkeit*) of the secretion indicates that it is uterine.

3. Milk-white secretion, that is, not viscid or filiform, denotes simple vaginal catarrh.

4. A puriform condition results from acute or chronic gonorrhœa, but may also depend upon other conditions. In the first case the urethra and Duverney's glands are especially affected, but not so in the others.

5. The microscope enables us to distinguish whether the discharge proceeds especially from the urethra or the vagina, and also whether it consists merely in an increased separation of epithelial cells (simple catarrh), or is accompanied by the formation of pus cells (blenorrhœa).

6. The author has carefully sought for, without ever finding, the trichomonas vaginalis, which Donné states to be characteristic of infecting flour albus; but the changed epithelial cells, enclosed in tough mucus, bear a great resemblance to the figures he has given.—(*Monatsch. fur Geburtsk.* Band V. 459.) *London L.*

